# Voyages in Vacation.—VI

**Published:** 1888-11-03

**Authors:** 


### Voyages in Vacation.—VI.

By One in Search of Restful Restoratives.

(*Continued from page 55.*)

### VI.—THE ADVANTAGES OF SEA TRAVEL.

If we hear persons declare that they always are fortunate in the weather when they go out in search of enjoyment or pleasure, we are apt to regard them as belonging to a tiresome class of folk in whom the *ego* crowds out every other consideration. When we look a little deeper into the question of weather, however, we soon find that the egotistical quality in man is distinctly helpful to him in many ways. The optimistic pleasure-seeker often owes his optimism to the discovery that by taking a little trouble he can pretty well forecast the weather, at certain places, at any time of the year. Then by selecting one which is generally bright and comfortable at the season he chooses for his holiday, he does in reality find Queen's weather, almost always, wherever he goes. We have said already that fine weather is one of three essentials to real enjoyment at sea. This no one will dispute, and so everybody should take care to arrange his yachting cruises to various places according to the climate and temperature there at particular periods of the year. Managers of the pleasure yachts are fully alive to this fact, and anyone who refers back to the second article* of this series, where several cruises and routes are given, will realise at once that the time selected for the visit to each country is governed by the very circumstances to which we here call attention. Those travellers who make out their own routes, and who prefer to go by the mail steamers, may be glad of our hint, however, for if they do not act upon it, they are sure to repent it bitterly. The s.s. *Garonne* (Orient Line, 5, Fenchurch Avenue) leaves London for a month's cruise in the Mediterranean, for example, on the 15th inst.

Those who travel much by railway know only too well the miseries of dust, and sun, and smoke. If the windows are kept closed, the dust not only penetrates, but the heat often becomes unbearable, and so first the ventilators and then the windows are opened. In a short time, the passenger finds his throat and mouth dry, his skin heated and sticky from the dust and warmth, and his temper (?)—well the less said about that the better. We believe there are few persons who can go through a long railway journey, except perhaps in wet weather, without experiencing an amount of discomfort which makes them hesitate to undertake it unless compelled to do so. In the winter, even in our temperate climate, some means of heating the sleeping carriages is found to be necessary, whilst abroad the temperature is forced up to a height which reduces ordinary people to a condition of helpless misery. In the regulation first-class carriage, the draughts are sometimes so bad as to be inimical to the comfort and dangerous to the health of the passenger. In a third-class or even more certainly in a second-class carriage the breezes which circulate up and about a passenger's legs are, as Mrs. Brown once put it, "something cruel." We do not, of course, deny that on the good lines and by the best trains all these evils are neutralised as much as possible. No doubt the journey from King's Cross to Edinburgh by the East Coast route is as comfortable a piece of travelling as one can meet with anywhere in the world. It is not too much to say that the Scotch express trains by the East Coast route leave absolutely nothing to be desired. Indeed, anyone possessed of the time and means ought to make an early opportunity of going to Edinburgh by this route, in order to see what the perfection of railway travelling in reality means. Still, as we said at the commencement, no management, however good, can prevent the presence of penetrating dust-showers which cover and smother the traveller to his increasing annoyance and disgust. Sea-sickness is almost, if not quite, preferable to the dust nuisance, and so even for bad sailors a pleasure yacht or well-found liner has many charms which the railway cannot offer.

Again, take the advantages which a steam yacht has over the railway abroad in matters of passport and Custom House regulations. Those who have had to cross the frontiers of France and Germany, and then to pursue their way to Russia, will understand the immense advantage of taking all such matters wholesale, as one does in a steamer. For Custom House purposes passengers are cargo, and so get passed through with the goods in bulk, rather than in detail, seeing that each passenger does not individually go through any ordeal by himself, but is only treated as one of sixty or a hundred, or whatever the number may be. Thus at Cronstadt, as will later appear, the passengers by the *Victoria* had simply to hand their passports to the purser, who afterwards handed them back endorsed, and thenceforth found themselves free to wander about Russia as they pleased. If the passport was ever asked for afterwards at all, which was improbable, the Cronstadt *visé* sufficed to satisfy the most exacting official. In the same way with luggage. Once on board the yacht, everything could be unpacked and put away in the presses provided in each cabin; the portmanteaus and trunks were consigned to the baggage-room, and for the whole trip caused no further trouble nor anxiety. This in itself is a great gain, and adds not a little to the advantages of sea travel. Then again, the *Victoria* has a carefully selected agent at each port of call, who is able to give the latest and best information about hotels, travelling, amusements, places to see, orders of admission, &c. Besides, the passenger's letters are sent to the agent's office in advance, and so by a little management can be brought by the agent when he first boards the yacht, and thenceforth day by day till the vessel sails. Should a letter arrive late the agent will forward it at once, and ensure its delivery before the next port is left. This is only one of many illustrations which might be given of the advantages of sea travel to all who have sufficient adventuresomeness in their constitution to desire to see the world, and so to make their lives as enjoyable and profitable as possible. If you were to ask one who has just returned from a sea trip what is the most comfortable thing about a well-found ocean-going steamer, you would probably find that you had given your friend a poser. You would generally hear by way of answer that everything was very nice except perhaps one horrid circumstance, which had hit the weak spot of the questioned. For instance, two ladies, sisters, of highly proper and religious tendencies, were much alarmed when a storm arose and shook the vessel, as storms unfortunately will, from stem to stern. Growing greatly alarmed, one of them went to the captain in genuine distress, and asked if they could not do something to help the ship. Might she not have her piano thrown overboard to lighten the vessel? When the captain assured her that there was no need for any such sacrifice on her part, and that it would be useless to so try to effect the object she aimed at, the lady replied: "Oh, Captain! Captain! what must we do?"

Captain: "Trust in providence, and all will be well in the end."

Lady Passenger (terrified): "Dear me! Captain, has it really come to *that?*"

Of course, there was no real danger of any kind, but when once the passengers' fears are excited they sometimes lose

No. 106, vol. v., page 7.

their heads, and then even religious people may make their religion, as in this case, an additional stimulus to their fears.

Where the impressions gained on a sea voyage are so mixed as they often undoubtedly are, no doubt it is difficult for a passenger, when challenged, to state clearly what feature of ocean travel he or she found most enjoyable. If the weather was cold, probably the average Britisher would say promptly, the open fire in the saloon, for they do have open fires on board some of the largest and best-found steamers, as many may be astonished to hear. Perhaps, then, the best way to bring all the comforts home to the reader will be to sketch briefly what the *Victoria* offered. First and foremost she had the electric light everywhere throughout the vessel, and the electricity was not stored in accumulators, but supplied direct by the dynamo which generated it. Yet the light given was always of the steadiest and best kind, and not once during the whole five weeks did the light flicker or fail in any way. Why cannot we have it so supplied to our houses at home? Will some electrical engineer kindly explain this riddle. Then each cabin was furnished with spring mattresses, separate and most excellently-planned wash-hand basins, bookshelves, holdalls, couches, a life-belt, both an electric and also a candle lamp, chests of drawers, and even a medicine measure, this last, by the way, horribly suggestive of the self medication, mark this, oh *Lancet*, in which some—must we conclude most—passengers indulge? A capital smoking-room, two excellent decks on which to take abundant exercise, and a saloon, encased in marble, and so admirably decorated as to make it one of the prettiest and most attractive to be found afloat. The library contained some very readable books, the officers were companionable and most obliging, the sailors intelligent and under excellent discipline. There was a choice of upper-deck cabins, where the portholes could almost always be kept open, and baths numerous and luxurious. Add to these comforts, deck chairs and awnings so arranged as to protect from the wind as well as from sun and wet, and we are inclined to think the *Victoria* is entitled to rank as a first-class floating hotel, which, with good administration, must meet the requirements of the oldest and most fastidious traveller. It may well be imagined that a fair sailor, when seated on the deck of such a steamer on a fine bracing day, was wont to say and to feel that nothing could be so health-restoring or more enjoyable, choose the whole world through.

(*To be continued.*)

